# Upcycling industrial peach waste to produce dissolving pulp

**DOI:** 10.1007/s11356-025-35977-5

**Published:** 2025-01-30

**Authors:** Sofia Plakantonaki, Nikolaos Zacharopoulos, Miltiadis Christopoulos, Kyriaki Kiskira, Giorgos Markou, Lamprini-Areti Tsakanika, Georgios Priniotakis

**Affiliations:** 1 Laboratory of Design and Development of Innovative Knitted Textiles and Garments, Department of Industrial Design and Production Engineering, University of West Attica, 12244 Egaleo, Attica Greece; 2 Hellenic Agricultural Organization-DEMETER, Institute of Technology of Agricultural Products, Sof. Venizelou 1, 14123 Lykovrysi, Greece; 3 Laboratory of Inorganic and Analytical Chemistry, School of Chemical Engineering, National Technical University of Athens, Zografou Campus, Heroon Polytechniou 9, 15773 Athens, Greece

**Keywords:** Food waste, Peach, Extraction, Cellulose, Pulp, Textiles, Recycling

## Abstract

This study investigates the production of high-purity cellulose pulp from peach (*Prunus persica*) fruit wastes generated during the processing of a Greek compote and juice production industry. A three-step chemical process is used, including alkaline treatment with NaOH, organic acid (acetic and formic) treatment, and hydrogen peroxide treatment, with the goal of cellulose extraction and purification. A fractional factorial design optimized reagent levels, revealing the strong influence of NaOH concentration on α-cellulose content and degree of polymerization. The extraction yield ranged between 9.7 and 12.3%, and a yield of 11.6% was achieved under the optimal conditions (NaOH, 3%w/v; acid-to-solid ratio, 6 l/kg; H_2_O_2_, 0.5% v/v) of the factorial experiment. The resulting samples were further characterized by XRD, FTIR, SEM, and TGA techniques. The results of the XRD and FTIR spectra confirmed the presence of cellulose, with a crystallinity index of approximately 57%, balancing strength and reactivity. The SEM analysis demonstrated a strong morphological similarity between the extracted pulp and commercial dissolving-grade pulp (Södra Purple), while images of the raw material confirmed effective purification. Additional evaluations included color, lightness, and lignin content. These findings suggest that cellulose extracted from peach waste is suitable for textile applications, offering a sustainable alternative to conventional cellulose sources and supporting industrial resource efficiency.

**Graphical Abstract:**

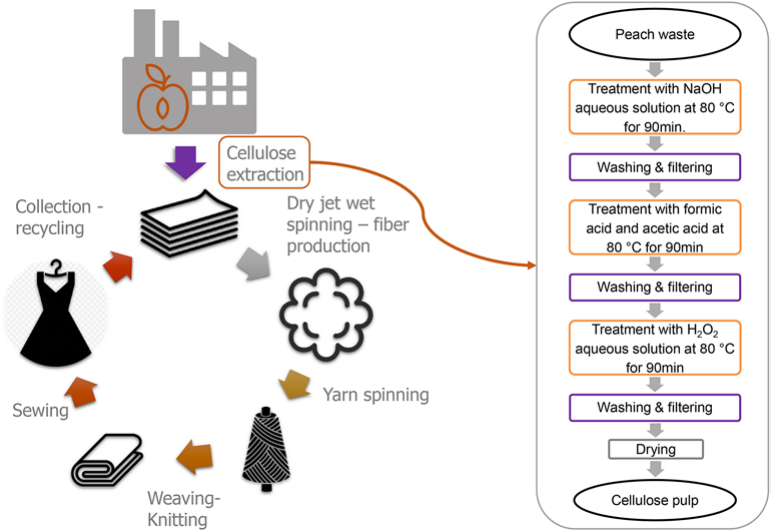

## Introduction

Food waste is generated as a result of the combination of high production rates and inadequate handling systems. The global average for food loss and waste is approximately 30% for cereals, 40–50% for root crops, fruits, and vegetables, and 20% for oilseeds, meat, and dairy (Liu et al. 2023). Furthermore, globally, agriculture produces over 6.3 Pg of dry matter annually in the form of organic waste products (Plakantonaki et al. 2024). The loss of natural resources has a significant environmental impact, leading to elevated levels of carbon dioxide emissions, contamination of soil and water, and shortages of food. Specifically, the worldwide peach (*Prunus persica*) harvest surpasses 25 million tons on a yearly basis, according to the FAO’s 2023 report (FAO 2023). The Union of Canners of Greece reports that around 60% of the entire peach crop is allocated to the food processing business. Substantial quantities of peach fruit waste are generated during the industrial processing of peaches, particularly when extracting peach juice or peeling and removing the pits to prepare compote. Developed countries discard around 35 to 40% of the waste produced from peaches after undergoing industrial processing (Hussain et al. 2020). Moreover, the quantity of peaches produced is influenced by their maturity, ripening stage, and the processing they undergo. The peach residue consists of skin (exocarp), fragments of fruit (mesocarp and exocarp), pomace (mesocarp and exocarp), pits (endocarp), and seeds.

The residual pulp and peels collected during the process of extracting peach juice contain a dietary fiber concentration of 54% (Pagán & Ibarz 1999). Fibrous applications stand out among various waste valorization options due to their significant potential for high value addition (Plakantonaki et al. 2024). Dietary fibers are classified according to their water solubility as either soluble or insoluble (Plakantonaki et al. 2023). Different types of dietary soluble fibers exist, including pectin, gums, and mucilage (found in aquatic plants, aloe vera, okra, and glycoproteins from food additives). Conversely, insoluble dietary fibers include cellulose, hemicellulose, and lignin. Dietary fibers, both soluble and insoluble, can be extracted via dry and wet processing, enzymatic, biological, and chemical methods, and also with new green methods such as ethanol extraction, ultrasonic-assisted extraction, and other combined techniques (Maphosa & Jideani 2016). According to Toushik et al. (2017), in the case of peach pomace, cellulose accounts for 28.7–30.0%, hemicellulose for 18.6–20.0%, and lignin for 5.35–6.0%, and the fibers can be extracted using various methods, such as chemical and enzymatic processes (Grigelmo-Miguel et al. 1999). On the other hand, peach pit contains a significant amount of lignin and a little amount of cellulose and comprises approximately 7% of the fruit’s total weight. Due to this reason, the core is not regarded as a reliable source of cellulose. However, it is commonly used as a biofuel, particularly in industrial facilities.

**Fig. 1:**
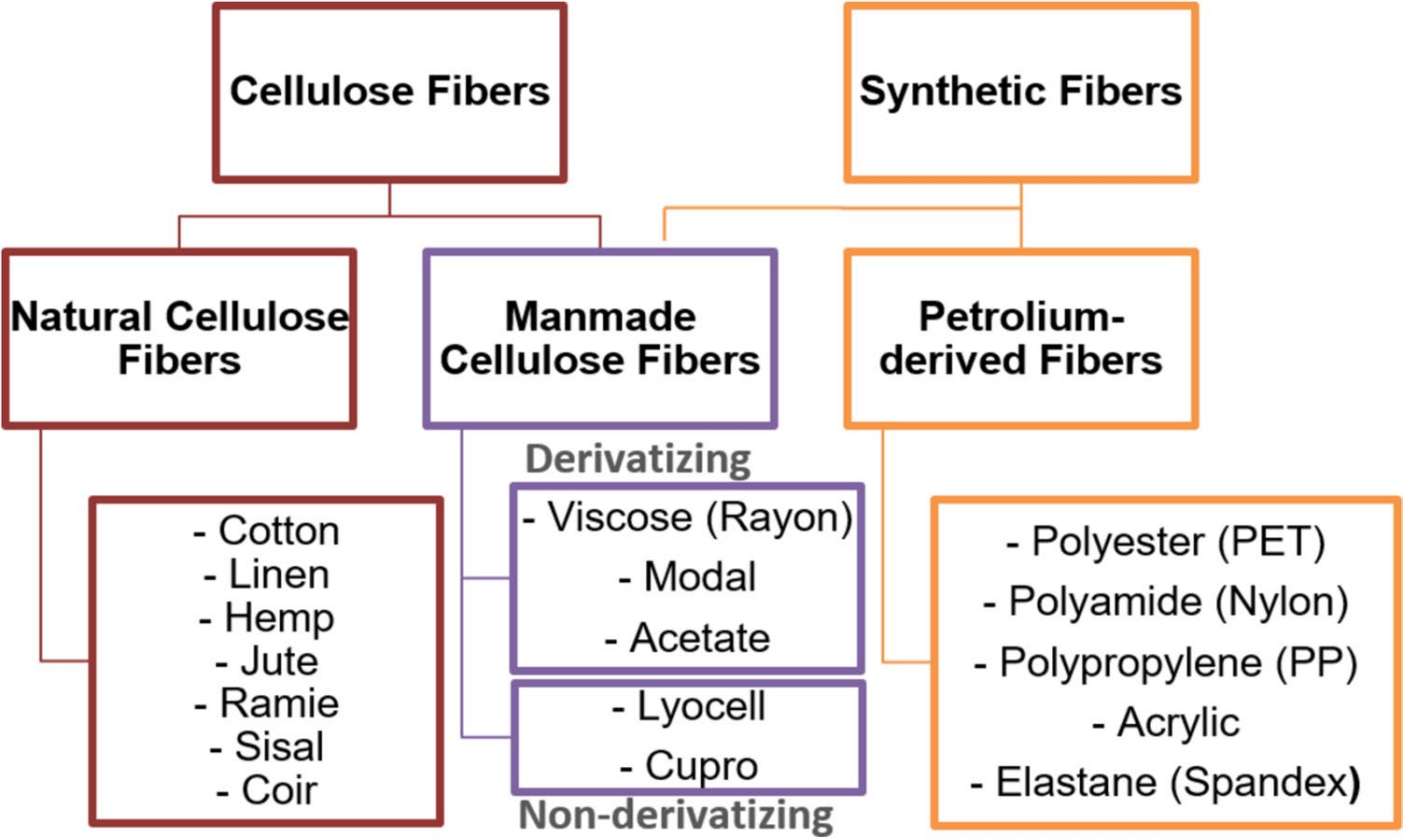
Categorization of conventional cellulose and synthetic fibers

Textile fibers are categorized into two distinct groups: natural and synthetic, as presented in Fig. 1. Humanity has been utilizing natural fibers for numerous centuries, which can originate from either plants or animals (Sayyed et al. 2019). Nevertheless, the exponential expansion of the textile sector during the twentieth century could not be adequately supported solely by natural fibers due to their seasonal production and restricted availability. Thus, synthetic fibers have been constantly gaining in market share, reaching a total of 71.3% in 2022, including manmade cellulosic fibers (MMFs) with a market share of 6.3% (Textile Exchange 2023).

MMFs, also known as regenerated cellulose fibers, are derived from dissolving pulp. Dissolving pulp has a cellulose content that is notably higher, ranging from 90 to 98%, compared to paper pulp, which contains 90% or less cellulose. Additionally, dissolving pulp has a low hemicellulose content (< 4%) and contains traces of lignin and resin (Ajao et al. 2018; Quintana et al. 2024). The state of the art in cellulose fiber production has predominantly focused on wood-based sources for dissolving pulp, employing and optimizing established chemical processes such as the kraft and sulfite methods (Balkissoon et al. 2023; Mendes et al. 2021; Quintana et al. 2024). Specifically, around 85% of the worldwide pulp output is derived from softwood species like fir and pine, as well as hardwood species like beech and eucalyptus. The remaining 10% is sourced from cotton linters, which are waste materials generated during the ginning process (Sixta 2006). The dissolving pulp obtained from these sources exhibits a cellulose content of over 90%, a consistent distribution of molecular weights, and a notable level of brightness.

**Fig. 2:**
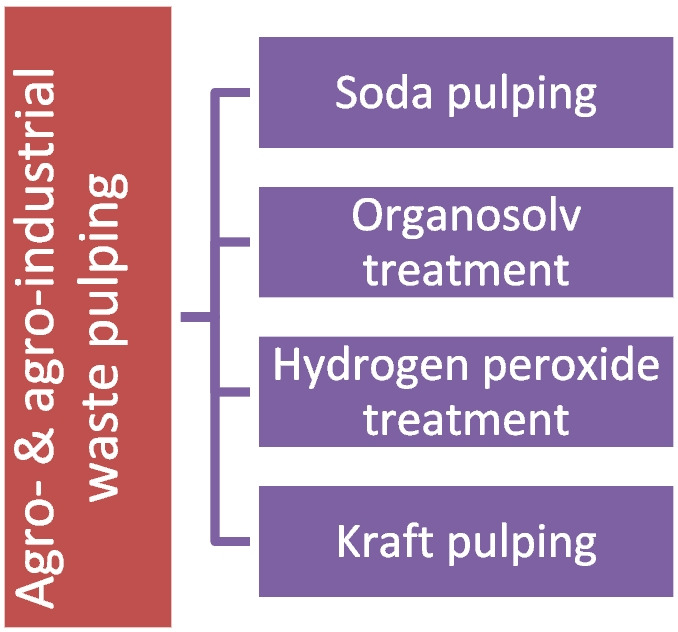
Methods to generate dissolving pulp from agro- and agro-industrial wastes

In recent years, there has been a significant amount of research conducted on the synthesis of pulp from non-wood plant sources (Plakantonaki et al. 2022), such as bamboo pulp, which is sold commercially (R. Batalha et al. 2011). By further exploring this path, utilizing lignocellulosic agricultural by-products has the potential to enhance pulp output and expand the market share of bio-based textile goods without necessitating more agricultural land for industrial crops or contributing to deforestation. A variety of chemical methods and combinations of processes have been researched by scientists to develop pulp from agro- and agro-industrial wastes, such as citrus fruits, borassus fruit waste, sugarcane bagasse, argan press cake, and cornhusk (Plakantonaki et al. 2022). However, no established way to produce cellulose pulp from peach waste is detected within the literature. Moreover, only a limited part of these studies targets the production of high-purity pulp for the textile industry. The major processes that have been utilized for this purpose are depicted in Fig. 2, whereas Table 1 includes recent research efforts to exploit these methods in various agro- and food-waste materials to produce high-purity pulp for the textile industry.

**Table 1:** Dissolving pulp from agro- and agro-industrial waste in research

Source	Process description	Final product	Reference
Coconut mesocarp (*Cocos nucifera*)	Multistage chemical treatment with NaOH, NaClO_2_, HNO₃, H_2_O_2_, CH_3_COOH, H_2_SO_4_, and centrifugation (max T = 120 °C, *t* > 18 h) → nanofibrils	Sewing thread via electrospinning	Rojas-Valencia et al. (2018)
Hemp shives/isolated fibers	Soda pulping, washing with CH_3_COOH, bleaching with H_2_O_2_ (max T = 170 °C, *t* > 2 h) *Hemp pulp (shives based): a-cellulose content: 87%, DP: 559* *Hemp pulp (fiber based): a-cellulose content: 96%, DP: 656* *Oil flax pulp: a-cellulose content: 91%, DP: 542*	Lyocell fiber	Kosan et al., (2024)
Oil flax fibers			
Jute wastes	Multistage treatment with HCOOH, NaOH, ClO_2_, H_2_O_2,_ (max T = 107 °C, *t* > 8.5 h) *a-cellulose content: 96%*	Dissolving pulp	Sarkar et al., (2018)
Jute stick	Pre-hydrolysis kraft pulping, multistage bleaching with ClO_2_, NaOH, H_2_O_2_ (max T = 160–170 °C, *t* = 3.5–4.5 h) *a-cellulose content: 92%*	Dissolving pulp	Matin et al., (2015)
Oil palm empty fruit bunch	Pre-hydrolysis, soda-anthraquinone (AQ) cooking, and elementary chlorine-free or totally chlorine-free bleaching (max T = 160 °C, *t* = 4.5 h, excluding bleaching) *a-cellulose content* > *95.9%*	Dissolving pulp	Harsono et al., (2016)
Oil palm whole-empty fruit bunches and stalk-empty fruit bunches	Pre-hydrolysis, soda cooking, and multistage bleaching with ClO_2_, H_2_O_2_, oxygen (max T = 165 °C, *t* = 13 h) *a-cellulose content* > *94%*	Dissolving Pulp for viscose	Nikmatin et al. (2022)
Orange	Multistage chemical treatment with toluene/ethanol, H_2_O_2_, CH_3_COOH/HCOOH, NaOH (max T = 90–110 °C) *Alpha-cellulose content* ≥ *90% w/w, yield* ≥ *10%*	Dissolving pulp	Santanocito and Vismara, (2015)
Sugarcane bagasse	Treatment with 10% NaOH (*T* = 90 °C, *t* = 24 h)	MMCF via wet spinning	Ma et al., (2021)
	Soda pulping 0.1% AQ (170 °C, 1.5 h)		
Wheat straw, switchgrass, and hemp hurd	SO_2_–ethanol–water pulping, extraction with NaOH, bleaching with ClO_2_ (max T = 130 °C, *t* = 6 h) *a-cellulose content: 93%*	Dissolving pulp	Vera et al., (2023)

*max T* the maximum temperature identified within the processes, *t* total time to complete the purification

In particular, organosolv processes stand out as an efficient alternative to conventional pulping methods for non-wood feedstocks (Jahan et al. 2021). They can support small-scale facilities and simplify chemical recovery via distillation (Huang et al. 2019; Rousu et al. 2002). Organosolv processes can produce high-quality, easily bleachable dissolving pulp and retain carbohydrates in the liquor, enabling monosaccharide recovery for biorefinery applications such as biofuels and green chemicals (Blair & Mabee 2021; Plakantonaki et al. 2024; Vera et al. 2023). They are also applicable in diverse feedstocks with consistent performance, highlighting their adaptability (Rousu et al. 2002; Vera et al. 2023).

In line with the selected production process of the manmade cellulosic fibers and the desired characteristics of the produced regenerated cellulosic fibers, different raw material (pulp) specifications may apply. Of particular interest is the lyocell method, which serves as a substitute for viscose in the production of regenerated cellulosic fibers (Opperskalski et al. 2022; Sayyed et al. 2019). In this process, cellulose is dissolved in an aqueous solution of N-methylmorpholine N-oxide (NMMO) without the need for harmful solvents. NMMO is recognized as an environmentally friendly solvent (Jiang et al. 2020) and may be recycled up to 99% in the lyocell process. The lyocell method is gaining popularity in commercial use due to its high-quality fabric and environmental benefits, and it is now the third most commonly used manmade cellulosic fiber, with a market share of approximately 4% in this category (Textile Exchange 2023). Important parameters for dissolving pulps are as follows:

### α-cellulose

Dissolving pulps have an α-cellulose content equal to or greater than 90% (Ajao et al. 2018; Quintana et al. 2024), whereas dissolving pulps for lyocell are typically purer with α-cellulose over 92% (Zhang et al. 2008). α-cellulose is used to describe the true cellulose fraction, which is the high-molecular-weight, crystalline component that is insoluble in alkali and water. On the other side, β- and γ-cellulose are associated with hemicellulose, with γ-cellulose being the more soluble and lower-molecular-weight fraction of hemicellulose. During the production of the spinning solution, the hemicelluloses in the pulp can also be dissolved in the aqueous NMMO solution; however, an excessively high amount of hemicellulose can reduce the mechanical strength of lyocell fibers (Jiang et al. 2020).

### Degree of polymerization (DP)

The degree of polymerization (DP) in cellulose is heavily influenced by both the source and the process used for extraction (Quintana et al. 2024). Pulps characterized by low DP values demonstrate higher cellulose dissolution. At the same time, the DP of the dissolving pulp has a direct impact on the mechanical strength of the fiber, with higher DP theoretically corresponding to greater mechanical strength of the fiber. As a result, optimizing the equilibrium between DP and pulp solubility significantly affects the spinning process and the resulting fiber’s performance. According to Jiang et al., DP values for lyocell between 650 and 750 may be considered better, with the intrinsic viscosity ranging from 280 to 350 mL/g (Jiang et al. 2020). Concurrently, Biswas et al. set the DP values for lyocell dissolving pulps between 500 and 900 (Biswas et al. 2021). Dissolving pulps for viscose fiber requires a pre-aging stage to reduce the viscosity to around 200–300 mL/g (Quintana et al. 2024).

Cellulose I is the form found in nature, present in green plants, algae, tunicates, and some bacteria (Zanchetta et al. 2021). Cellulose I can undergo irreversible transformation into a stable crystalline form known as cellulose II (PÉrez & Samain, 2010). Mercerization and regeneration are used in the textile industry to accomplish the transformation into this allomorph. The process of immersing cellulose in a concentrated aqueous solution of sodium hydroxide (NaOH) mercerizes the cellulose, leading to swelling within the crystal structure and subsequent recrystallization upon washing. Regeneration takes place following the process of coagulation and recrystallization of a solution containing a cellulose or cellulosic intermediate product. Cellulose I is characterized by a parallel alignment of cellulose chains, while cellulose II is characterized by an antiparallel alignment. Cellulose I or II can be transformed into cellulose III by chemical treatment using ammonia or certain ammines. Subjecting cellulose III to high temperatures and treating it with glycerol lead to the formation of cellulose IV.

In the research for new environmentally friendly, biobased, and biodegradable raw materials to meet the needs of the textile industry, the possibility of processing peach wastes from the food industry (compote and juice production) to develop dissolving pulp for regenerated cellulosic fibers is researched. The objective is to upcycle the easily accessible agricultural waste of peach pomaces and produce dissolving pulp with high cellulosic content by implementing a three-stage treatment. This work examines for the first time in the literature the generation of cellulose pulp from peach fruit by-products and implements a novel three-step chemical procedure, comprising of existing well-established processes:

1. Alkaline treatment with NaOH: The addition of sodium hydroxide supports the removal of hemicelluloses from the waste material (Gehmayr & Sixta 2012; Sixta 2006). The mechanism can be divided into two successive stages: (a) physical interaction between cellulose and aqueous sodium hydroxide resulting in fibers swelling; (b) diffusion of hemicelluloses from the fibers interior to exterior via the swollen pores in the fiber wall and their transfer to the aqueous solution (J. Li et al. 2015).
2. Treatment with organic acids (acetic and formic): The solid that has undergone the swelling procedure during the first step of the process is now more susceptible to further purification to remove the unwanted substances. The use of organic solvents demonstrates the ability to remove lignin and hemicelluloses under mild conditions, without significant cellulose degradation. The rate of delignification in acidic conditions is controlled by α-ether cleavage, while the likelihood of β-ether cleavage increases in more strongly acidic systems (Lu & Ralph 2010).
3. H_2_O_2_ treatment: The application of H_2_O_2_ is thought to enhance the pulp’s delignification process. In basic solutions, hydrogen peroxide is a strong reductant, and oxygen gas is also produced. Sodium hydroxide acts as the solubilizing agent for extracting modified lignin fragments, while hydrogen peroxide and oxygen—that is, those produced from the decomposition of H_2_O_2_—attack and fragment the residual lignin (Llano et al. 2018). The same mechanism can remove hemicelluloses produced due to cellulose decomposition in the previous steps of the procedure. H_2_O_2_ bleaching is performed together with an alkali source to produce the active perhydroxyl anion and the perhydroxyl free radical that have been suggested as the intermediates in the brightening reactions for cellulose-based products (Brooks & Moore 2000; Zeronian & Inglesby 1995). Hydrogen peroxide decomposes to form water and oxygen, and as a result, no residues of the bleaching agent nor organochlorine by-products are found in the final product.

## Material and methods

### Raw material

**Fig. 3:**
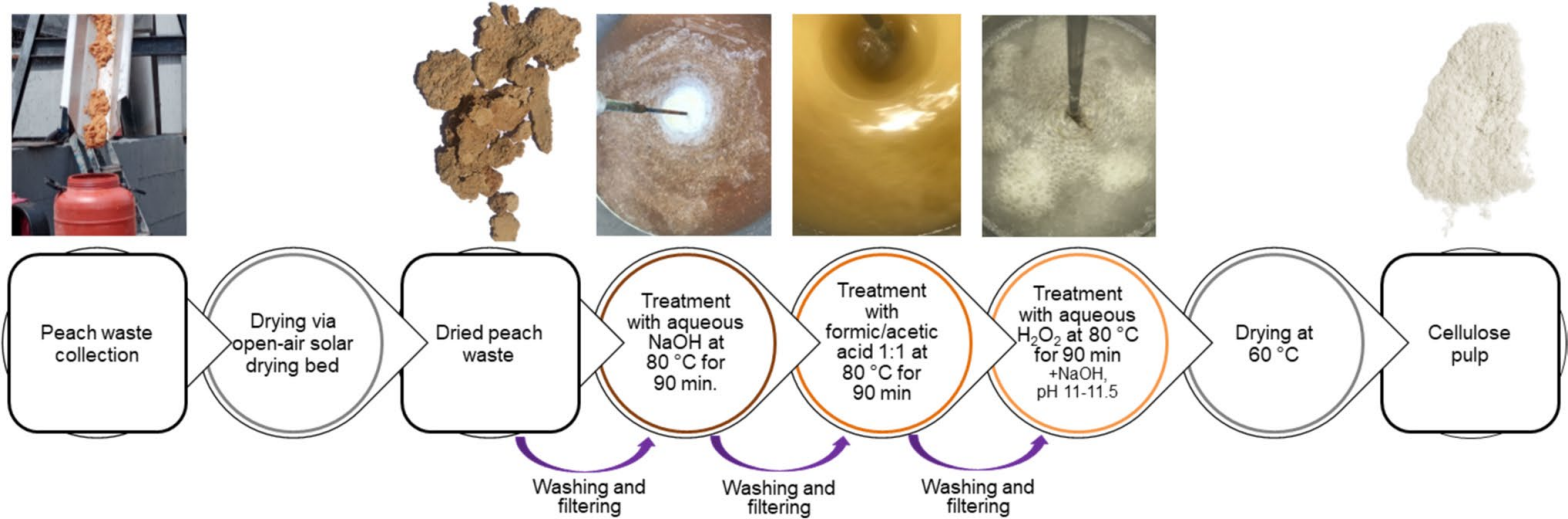
Schematic representation of the collection and processing steps for peach wastes to produce cellulose pulp

Peach (*Prunus persica* (L.) Batsch) wastes were obtained from the AGROPHEONIX industry in Imathia, Greece. The wastes consisted of residues from the compote and juice production lines, which included a mixture of peels and pomaces. They were collected in 60-L plastic cans (see Fig. 3), suitable for food storage, placed in an open-air solar drying bed to dry, and then stored in polypropylene bags at room temperature (20–25 °C) until the extraction process. The final humidity content was 9.59%.

### Chemical cellulose extraction and purification

The dried pitted peach waste underwent a three-step chemical process, with drying as the only pre-treatment.

A. One hundred grams of the dried pitted peach waste were mixed with an aqueous solution containing 1%, 3%, or 5% w/v NaOH with a solid-to-liquid ratio of 1:20 in an appropriate stainless steel container, which was heated at a temperature of 80 ± 2 °C under continuous mechanical stirring for 90 min. The suspension was filtrated via sieves (1.6 mm to 125 μm), and the precipitate was washed thoroughly with tap water until neutral pH.
B. The resulting solid from the first process was treated with a solution of formic acid 85% v/v and acetic acid 80% v/v mixed in equal proportions. The suspension was heated at a temperature of 80 ± 2 °C under continuous mechanical stirring for 90 min. The solid-to-liquid ratio was adjusted to 1:6 or 1:12 according to the design of the experiment (DoE). The suspension was filtrated via sieves (1 mm to 63 μm), and the precipitate was washed thoroughly with tap water until neutral pH.
C. The resulting solid was further purified and bleached with 20 volumes of H_2_O_2_ aqueous solution at concentrations of 0.5% v/v or 3.5% v/v containing 1 mL and 4 mL of silicone antifoaming liquid. NaOH was added to adjust pH levels between 11 and 11.5. The suspension was heated at a temperature of 80 ± 2 °C under continuous mechanical stirring for 90 min. Then, the suspension was filtrated via sieves (710 to 63 μm), and the precipitate was washed thoroughly with tap water until neutral pH. The cellulosic product was filtered via sieves, washed with tap water and acetone, and dried at 60 °C until stabilized weight.

Experiments took place at 1 atm. At the end of treatment steps A and B, large foreign matters and precipitates, such as partial peach cores, that were not extracted during the industrial peach processing were removed.

All reagents used in the pulping process were of technical grade and purchased from KALOCHEM A.E., except for the silicone antifoaming liquid that was purchased from AppliChem GmbH.

Figure 3 integrates a schematic and photographic representation of the three stages.

### Experimental design and analysis

To test the pulping ability of peach waste and assess the influence of the reagents used in each cooking bath on the parameters defining the progress of the pulping process, a fractional factorial DoE was conducted. DP and α-cellulose (%) content were selected as dependent variables to examine the ability of the method to produce cellulose pulp for textile use. The standard methods used to determine their values are described in the section on pulp characterization. A multistage DoE approach was selected by taking into consideration that measurements of both dependent variables require purified bleached pulp and that changes in one step might affect downstream steps of the process and the final product. Factors selected as independent variables were NaOH concentration (% w/v) in stage A, liquid-to-starting peach mass ratio (L/kg) in stage B, and H_2_O_2_ concentration (% v/v) in stage C. A three-level experimentation was selected for factor A: % w/v NaOH, as its pulping ability is also linked with the degradation of the carbohydrates at the end of the cellulose chain that can lower the DP (Q. Li et al. 2017; Sayakulu & Soloi 2022). For factor B: organic acids liquid volume-to-starting peach mass ratio (L/kg) and C: %v/v H_2_O_2_, two levels were implemented. The specific values of the levels of the independent variables used in the experimental design are presented in Table 2. Other variables, including reaction time and temperature, were kept constant. The average value of the replications for each dependent variable was used to perform the regression analysis using JMP® Pro (18.0.2) statistical software (SAS Institute Inc., 2024). The DoE consisted of six experiments and three replications in each experiment. Statistical tools and metrics used to assess the experimental results include the standard deviation of the response variable, regression models, root mean square error (RMSE), R-squared (*R*^2^), and *p*-values.

**Table 2:** Experimental design and results

No	Sample s	Factor A: %w/v NaOH	Factor B: acid to solid ratio (L/kg)	Factor C: %v/v H_2_O_2_	[η] (mL/g)	DP		α-cellulose (%)	
1	A1B1C1-S1	5	12	3.5	218.9	419	422 ± 5	82.5	82.3 ± 0.3
2	A1B1C1-S2	5	12	3.5	223.1	428		81.9	
3	A1B1C1-S3	5	12	3.5	218.5	418		82.4	
4	A1B3C3-S1	5	6	0.5	266.9	522	522 ± 3	84.7	84.4 ± 0.4
5	A1B3C3-S2	5	6	0.5	265.4	519		83.9	
6	A1B3C3-S3	5	6	0.5	268.1	525		84.7	
7	A3B1C1-S1	3	12	3.5	270.3	530	526 ± 5	94.3	94.2 ± 0.3
8	A3B1C1-S2	3	12	3.5	266.3	521		94.5	
9	A3B1C1-S3	3	12	3.5	269.6	528		93.9	
10	A3B3C3-S1	3	6	0.5	305.9	608	604 ± 4	95.7	95.4 ± 0.3
11	A3B3C3-S2	3	6	0.5	302.4	601		95.2	
12	A3B3C3-S3	3	6	0.5	303.2	602		95.2	
13	A4B1C3-S1	1	12	0.5	235.2	454	460 ± 7	86.5	86.5 ± 0.2
14	A4B1C3-S2	1	12	0.5	237.5	459		86.3	
15	A4B1C3-S3	1	12	0.5	241.7	468		86.7	
16	A4B3C1-S1	1	6	3.5	238.6	461	465 ± 6	83.3	83.7 ± 0.7
17	A4B3C1-S2	1	6	3.5	243.1	471		84.5	
18	A4B3C1-S3	1	6	3.5	238.8	462		83.4	

### Pulp characterization

Viscometry measurements were performed in cupri-ethylenediamine solution to determine the degree of polymerization (DP), following the international standard ISO 5351:2010. Two viscosity measurements were made in each sample, and the results of the limiting viscosity number [η] are presented in Table 1 as an average of the measurements. The determination of the number of monomer units in the cellulose molecule is done by first calculating the molecular weight (MW) by using the following correlation equation (Kim et al. 2001):

$$\lbrack\mathrm\eta\rbrack\:=\:0.98\:\times\:10^{-2}\times\mathrm{MW}^{0,9}$$

DP is measured by dividing the MW of the polymer by the MW of the cellulose monomer.

TAPPI standards were applied to determine α-cellulose, β-cellulose, and γ-cellulose in the resulting pulps (T 203 cm-99). Pulp yield was determined gravimetrically and expressed as g dried pulp/100 g dry initial peach waste (% dry basis) after oven drying until constant weight. ISO 302:2004 was applied to determine the kappa number of the pulp. The percentage of lignin in the samples was calculated based on the formula: lignin (%) = kappa number × 0.13.

Moisture content was determined gravimetrically after drying the material at 100 °C for 3 h and allowing it to come to room temperature.

To measure the color, the colorimetric method was used, specifically the differential colorimeter Minolta CR-300 (Minolta, Germany), which measures the CIE (Commission Internationale de l’éclairage) coordinates L*, a*, and b*. The parameter L* yields the lightness of the color, while the parameters a* and b* express the red-green and yellow-blue colors, respectively. Positive a* values indicate a color shift towards red, while negative a* values indicate a color shift towards green. Respectively, positive b* values indicate a color shift towards yellow, and negative b* values indicate a color shift towards blue. The L* parameter is used to quantify the visual perception of the luminance (Kingdom 2011), where 0 corresponds to black and 100 to white. Three measurements were taken on each sample.

The XRD measurements were carried out using powder X-ray diffraction (Bruker D8 Advance diffractometer, Bruker, MA, USA) with Cu K radiation of wavelength 1.54060 Å, a voltage of 40 kV, a current of 40 mA, and a lower discriminator of 0.18 V. The scanning range was from 10 to 70° with a 0.02 increment. For the sampling, a portion of each sample was ground without any medium in an agate mortar and placed in the holder placed on a Teflon support at the center of a Plexiglas ring. The sample was spread from the center to the edge. A lid was set on the top and pressed to spread the mixture evenly and create a flat, smooth surface in order to avoid self-orientation of the crystals without exceeding the height of the ring. Then the Plexiglas ring was placed on a metallic sample holder, which was placed in the sample position of the instrument, where it was magnetically supported. The evaluation of the diffractogram was carried out by DIFFRAC.EVA software.

Fourier transform infrared (FTIR) analysis was performed using a single-beam FTIR Jasco 4200 spectrometer with a TGS detector supplied by Jasco Corporation (Tokyo, Japan). The FTIR spectra of the samples were obtained in the spectral range from 4000 to 400 cm^−1^. A resolution of 4.0 cm^−1^ and 32 scans were also selected. All samples were pelletized using KBr of IR grade (221864, Honeywell, Charlotte, NC, USA) on powdered samples. Potassium bromide and the powdered samples were mixed at a ratio of 200:1, and the mixtures were homogenized and further pulverized using an agate mortar and pestle. Then they were pressed at high pressure of 10 tons with the help of a hydraulic pressing system. The FTIR spectra were performed in transmission mode, and they were processed using Jasco Spectra Manager software.

Scanning electron microscopy (SEM) was utilized to examine the purification process and microstructural alterations that occurred as a result of the multistage treatment of peach waste. The samples underwent metallographic preparation and gold plating in order to improve their electrical conductivity. The materials were examined using the JEOL 6300 scanning electron microscope at different levels of magnification to deliver the characteristic three-dimensional images.

TGA measurements were performed on a Mettler Toledo TGA/DSC 1 HT apparatus. During TGA analysis, the mass of a sample is continuously recorded in a controlled atmosphere as a function of temperature as it increases. The sample size is small (up to 50 mg) so that errors due to thermal components are avoided. The temperature range considered in the present study was 30–800 °C with a heating rate of 10 °C/min and in a nitrogen atmosphere flow of 10 mL/min.

## Results and discussion

### Evaluation of the chemical pulping treatment

In order to establish the suggested chemical process as plausible for the extraction and purification of fibrils from peach waste and screen the effect of the reagents in each step of the chemical pulping process on the major properties of the resulting cellulose pulp, a custom fractional factorial design was constructed and implemented, the results of which are presented in Table 1. Initial experiments demonstrated the incapacity of each single process step to perform the extraction and purification of cellulose fibrils, ending up with obvious impurities.

The α-cellulose (%) content was over 80% with samples A3B1C1 and A3B3C3, consisting of high-purified cellulose pulp with an α-cellulose content above 94%. Simultaneously, the same samples demonstrate greater values on the DP parameter measurements, with samples A3B3C3 having DP values above the threshold of 600.

It is also noticeable that the intermediate level of the independent variable of NaOH %w/v concentration in the initial step of the process renders the best results. By examining the responses received, it is suggested that overprocessing by using the upper levels of the reagents leads to the partial depolymerization of the peach waste cellulose macromolecules. The result is a lower DP and α-cellulose content in the final pulp. Conversely, when selecting the lower levels of the reagents, it is suggested that an incomplete purification process takes place, which does not remove all other ingredients of peach residues, including hemicelluloses. This also results in reduced DP and α-cellulose content (%).

**Fig. 4:**
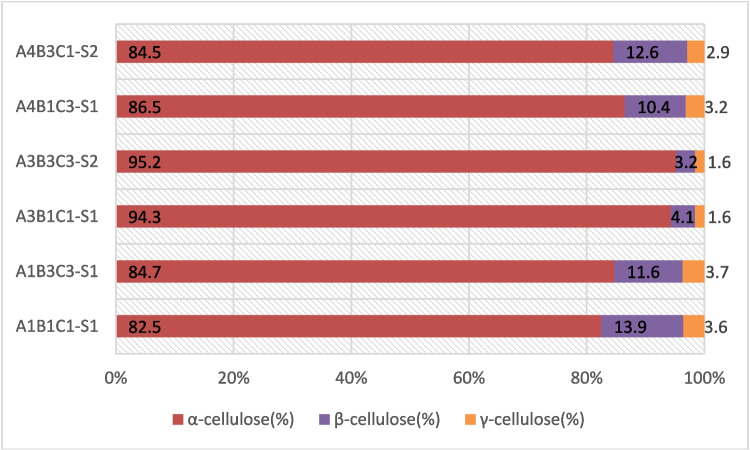
Graphical representation of % cellulosic clusters in selected samples

The percentage of each cellulosic fraction according to the TAPPI T 203 cm-99 method of analysis is presented in Fig. 4. β-cellulose is the soluble fraction in 17.5% NaOH solution, which is reprecipitated on acidification of the solution. γ-cellulose is the fraction that remains soluble in a 17.5% NaOH solution. γ-cellulose has the lowest molecular weight among the three types.

**Fig. 5:**
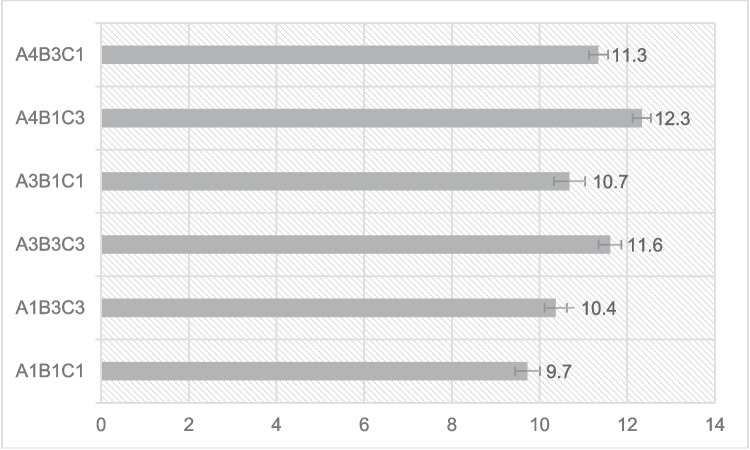
Pulp yield per sample

The overall yield of the three-step process is presented in Fig. 5. The upper levels of the reagents result in lower yields, suggesting a decomposition of the cellulose that is easily subtracted during the washing procedure from the pulp. On the other end, greater yields are produced when implementing the lower level of NaOH. These samples are lacking, though, in α-cellulose content when compared to the intermediate level of NaOH, which implies an incomplete subtraction of non-cellulose substances.

### DoE and statistical analysis

**Fig. 6:**
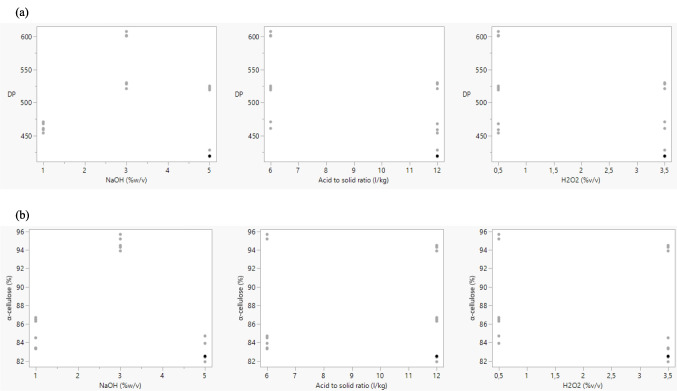
DP response (**a**) and α-cellulose (%) response (**b**) per factor and level

The bivariate relationship between dependent and independent variables is modeled in Fig. 6.

**Fig. 7:**
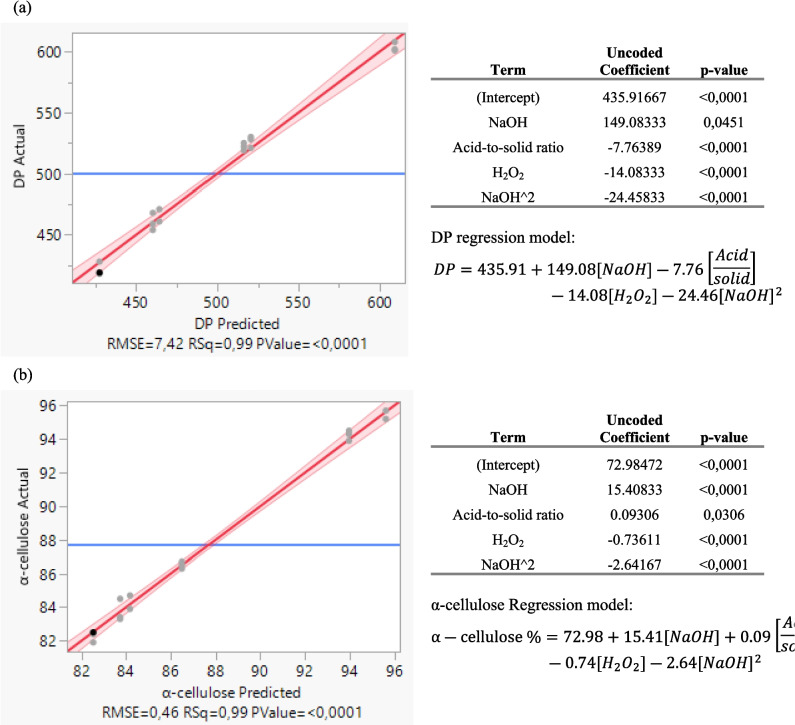
Regression model for each dependent variable DP (**a**) and α-cellulose (**b**)

Figure 7 shows the regression models for the DoE responses.

Statistical parameters demonstrate a great fit for both responses’ regression models (DP: R-squared = 0.99, RMSE = 7.42; α-cellulose: DP: R-squared = 0.99, RMSE = 0.46). The use of three levels in the experiments revealed an important second-order effect for the concentration of NaOH. Additionally, we observe that NaOH demonstrates the greater first-order coefficients in both regression models, displaying the larger effect in the responses. This observation can be attributed to the fact that it is used in stage A of the process, and thus it subtracts the greater amount of unwanted substances from the peach waste. All factors have a statistically significant effect with *p*-values less than 0.05. Nevertheless, by examining the coefficients of the regression model, the effect size of the acid-to-solid ratio (l/kg) and %v/v H_2_O_2_ appears to be rather low, with limited contributions in the response of DP and a-cellulose when the level changes. This reasoning guides the decision about the level used for these reagents on techno-economic grounds or other important parameters such as the final whiteness of the sample.

The experimental design allowed the investigation of interaction effects among the independent variables, including %w/v NaOH, acid-to-solid ratio, and %v/v H₂O₂. Nevertheless, the statistical analysis experienced challenges with accurately measuring certain interaction factors. The regression model demonstrated problems of multicollinearity and over-parameterization, leading to the inability to incorporate more than one interaction term without jeopardizing model stability. Furthermore, the uncoded estimates for the interaction effects, including %w/v NaOH × acid-to-solid ratio and %w/v NaOH × %v/v H₂O₂, were very small, indicating that these interactions exerted limited practical influence on the dependent variable. Based on these findings, the research concentrated on the main effects of the independent variables, which exhibited robust statistical significance and yielded valuable insights into the process.

### Color and brightness analysis

**Fig. 8:**
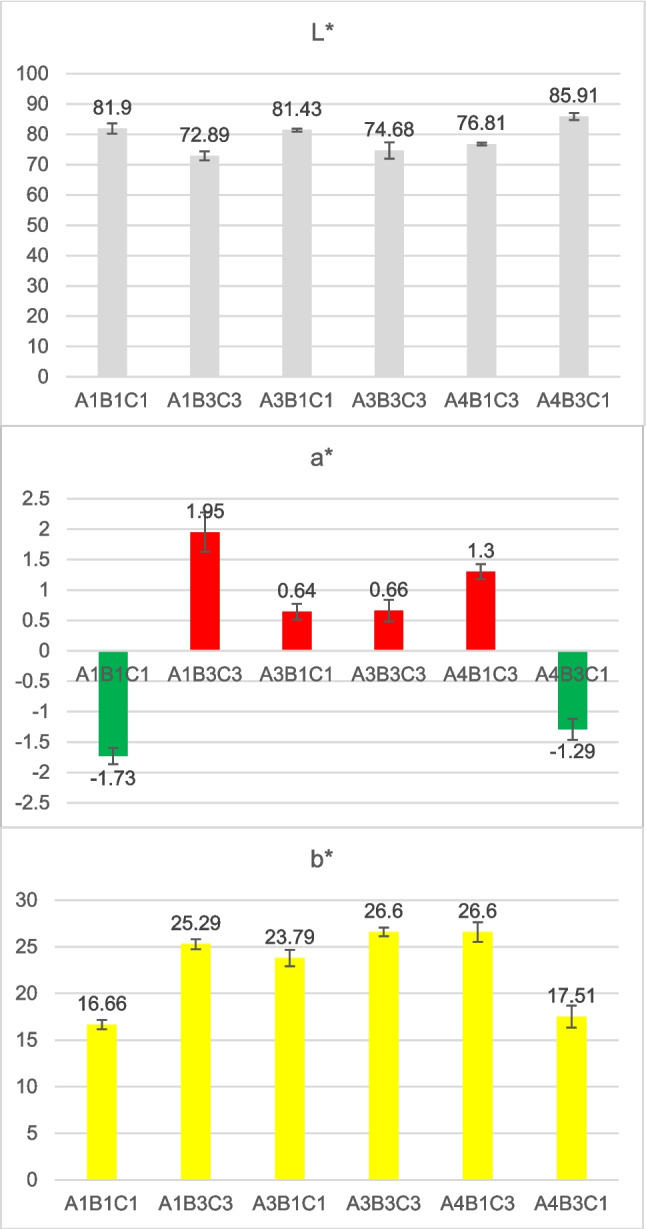
CIE L*a*b* coordinates of the treated pulps. The parameters L*, a*, and b* are shown in separate panels within the figure.

The proposed pulping sequence of the peach residues includes a final delignification and bleaching step that is performed via the use of an aqueous solution of H_2_O_2_ in an alkaline environment (11 > pH > 11.5). H_2_O_2_ can be employed to eliminate or whiten any remaining lignin and other chromophores that are responsible for discoloration. It is a more environmentally favorable chemical agent than chlorine-based alternatives since the decomposition of H_2_O_2_ leads to the production of oxygen and water, leaving no harmful residues (Suess 2010). Figure 8 presents the results of the CIE L*, a*, and b* coordinates.

The parameter L*, which represents the visual perception of an object’s luminance, is higher when the experiment is conducted with the upper level of H₂O₂, as expected. All samples have an L* value over 72, with sample A4B3C1 demonstrating a higher degree of lightness, which is equal to 85.91. Parameter a* received very low values, indicating that the pulp color does not shift towards red or green. However, the positive b* values indicate a color shift towards yellow. As a result, complete discoloration of the produced pulp was not possible under the experimental conditions. Achieving high whiteness of recycled pulps coming either from open-loop processes, such as from agro-wastes, or closed-loop processes post- or pre-consumer textile wastes is an engaging issue (Walawska et al. 2024) that requires further research.

### Kappa number and lignin

**Fig. 9:**
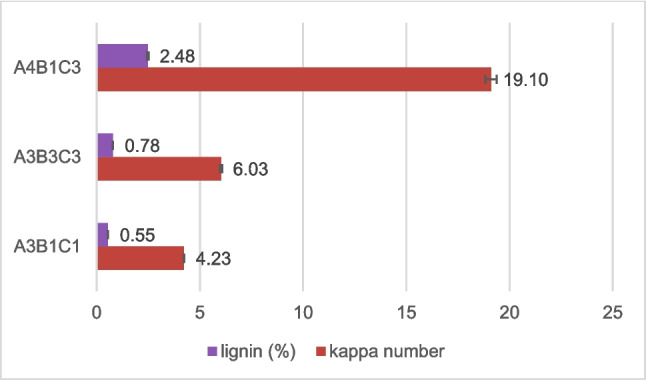
Kappa number and lignin

Samples A3B1C1, A3B3C3, and A4B1C3 that recorded the highest α-cellulose values were further examined for kappa number and the lignin content (%). Figure 9 illustrates the results.

All samples contained less than 2.5% lignin, with samples A3B1C1 and A3B3C3 demonstrating a lignin content of less than 0.8%. The higher content of lignin in sample A4B1C3 is associated with the lower levels of the reagents NaOH and H_2_O_2_ used during the process, resulting in decreased delignification.

### XRD analysis

**Fig. 10:**
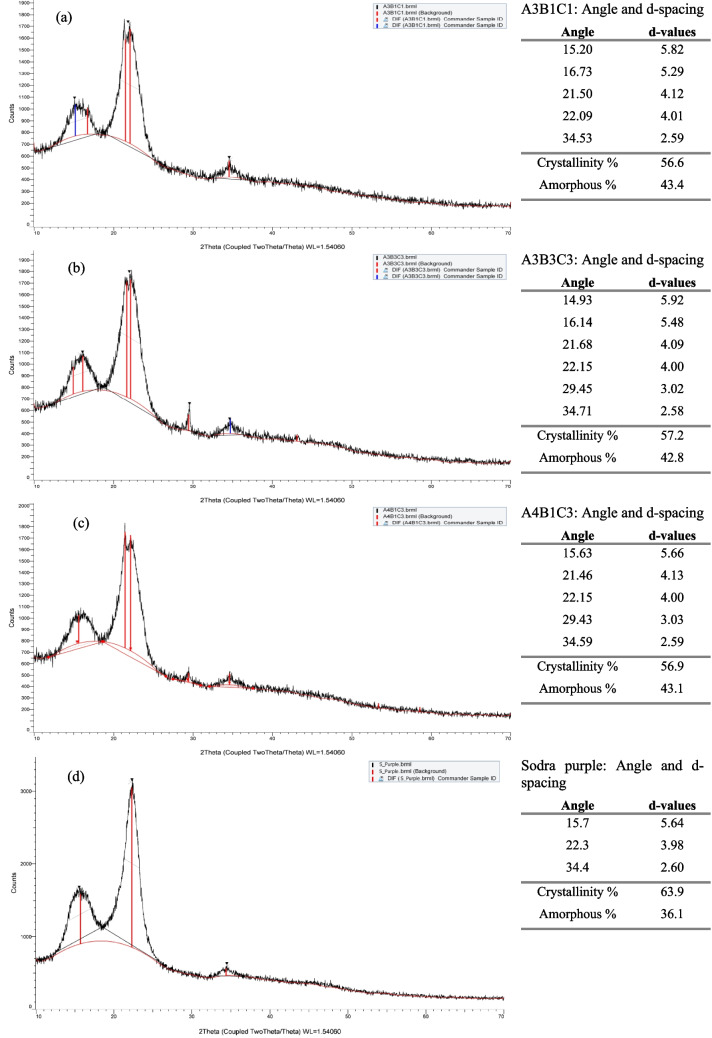
XRD patterns, angle, and d-spacing values for samples A3B1C1 (**a**), A3B3C3 (**b**), A4B1C3 (**c**), and Södra purple (**d**)

XRD has been employed for sample characterization and cellulose crystallinity analysis. A3B1C1, A3B3C3, and A4B1C3 samples were analyzed by XRD Ray crystallography, and the obtained results are presented in Fig. 10a–c. For comparison reasons, a commercial-grade dissolving pulp, Södra purple, was also analyzed for reference, and the results are presented in Fig. 10d.

In all samples, three well-defined crystalline peaks contribute to the diffraction pattern at approximately 15°, 22°, and 34.5° 2θ and are close to the typical cellulose I peaks (French 2014; Gong et al. 2017; Lee et al. 2013). In particular, several reflections combine to form the moderate peak near 34.5°. The d-values presented in Fig. 10 are in agreement with the literature, indicating the presence of cellulose (Chiriac et al. 2014). Additional peaks are identified at ~ 21° 2θ for samples A3B1C1, A3B3C3, and A4B1C3 and also at 29.4° 2θ for samples A3B3C3 and A4B1C3. According to the literature, the XRD pattern of cellulose II has diffraction peaks at ~ 12°, 20°, and 22° 2θ, corresponding to the (1 1 0), (1 1 0), and (0 2 0) planes, respectively. Based on these data, the peak determined at 2θ around 21° for A3B1C1, A3B3C3, and A4B1C3 samples can be attributed to the co-presence of cellulose II (Gong et al. 2017; Lee et al. 2013). The lack of a peak at about 12° 2θ¸ for cellulose II can be due to the low concentration of the substance as well as the presence of amorphous material, which moves the peak slightly. This displacement is overshadowed by the broad peak occurring between 13 and 17° 2θ. In the case of A3B3C3 and A4B1C3 samples, the peak observed at 29.4° 2θ may be attributed to the presence of xylan. In this case, the major peak of xylan, which is typically found around 2θ 21–22°, is covered by the dominating peak (Demma Carà et al. 2013). In Fig. 10d, the XRD pattern of the commercial dissolving pulp, Södra purple, is illustrated as a reference. Three well-defined crystalline peaks are contributing to the diffraction pattern at ~ 16°, 22°, and 34.5° 2θ, which are close to the typical peaks of cellulose I.

The crystallinity of the samples was automatically calculated by XRD Bruker D8 Advance. All samples presented insignificant differences, and the crystallinity degree ranged between 56.6 and 57.2%. High-crystallinity fibrils in pulp create a compact structure that limits the accessibility and reactivity of the pulp and challenges its dissolution to produce the spinning dope (Quintana et al. 2024). High crystalline pulps tend to have better mechanical strength but are harder to dissolve, while less crystalline pulps are easier to dissolve, more reactive, and more suitable for chemical processing. Södra purple was measured to have 63.9% crystallinity. Moreover, according to Mendes et al. (2021), commercial pulp from hardwood demonstrates a crystallinity degree of 54% when produced with acid sulfite pulping (ASP) and 56% when pre-hydrolysis kraft (PHK) pulping is used.

The XRD analysis confirmed the dominant presence of cellulose through consistent patterns and bands. In addition to cellulose I, strong evidence revealed the formation of cellulose II, attributed to the NaOH treatment used for cleaning and extraction. The crystallinity percentage, ranging between 56.6 and 57.2%, reflects a well-achieved balance: higher crystallinity enhances mechanical strength, while lower crystallinity improves reactivity and dissolution capacity.

### FTIR analysis

**Fig. 11:**
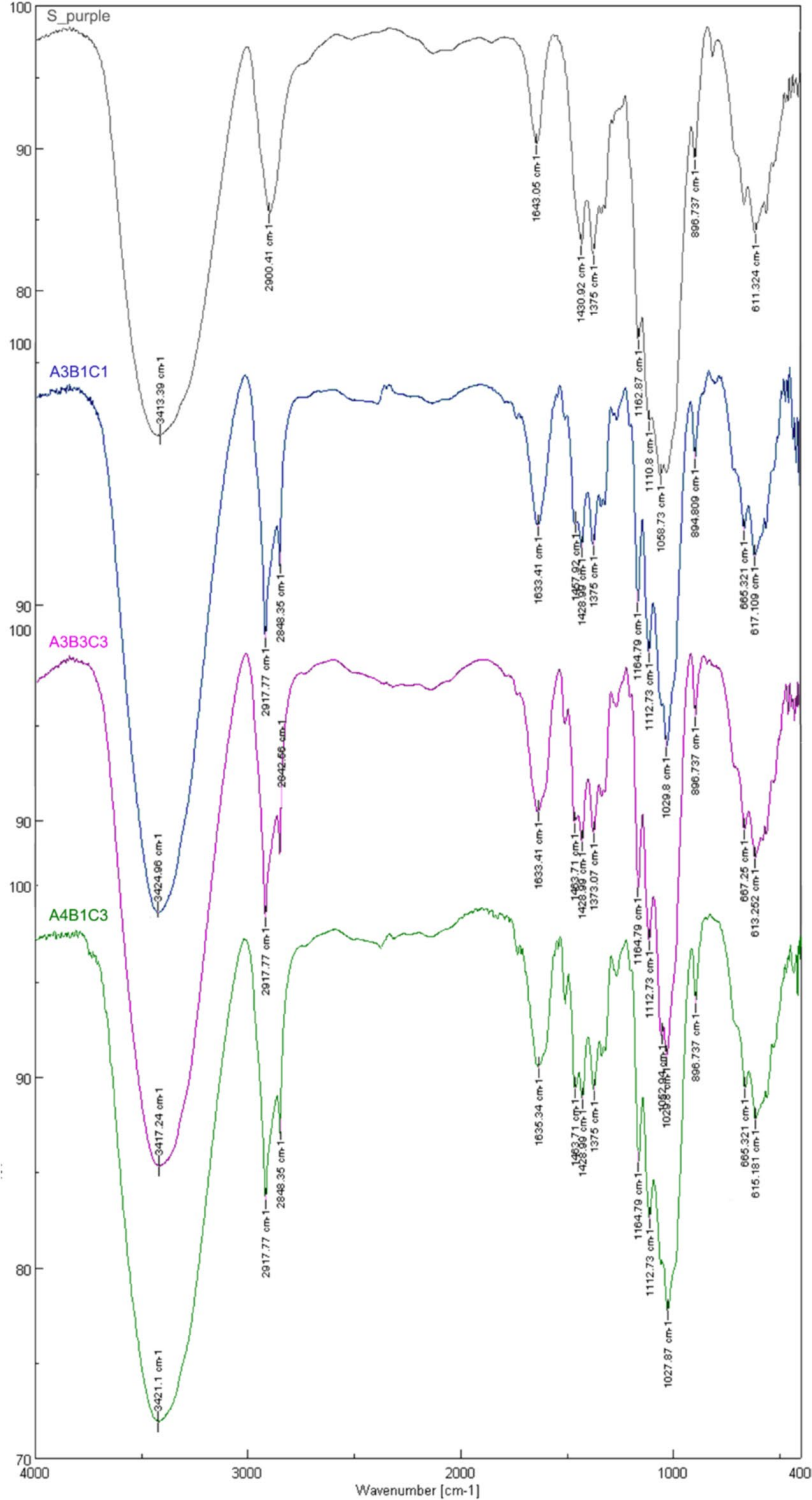
FTIR spectrum for the samples A4B1C3 (green), Α3Β3C3 (magenta), Α3C1B1 (blue), and Södra purple (grey)

The structural composition of the experimental pulps was confirmed using the FTIR analytical technique. Figure 11 illustrates the FTIR spectra obtained from the investigated samples A3B1C1, A3B3C3, and A4B1C3. The same figure depicts the FTIR spectrum for commercial-grade dissolving pulp, Södra purple.

Cellulose shows bands at IR spectra due to [ν(O–H)] stretching, [ν(C–H2)] stretching, [δ(C–H)] bending, [ν(C–O–C)] pyranose ring vibration, and β-glycosidic linkage between the glucose units in cellulose, respectively. In the range of 3660–2900 cm^−1^, the peaks are characteristic of the stretching vibration of [ν(O–H)] and [ν(C–H)] bonds (Dritsopoulos et al. 2024). More specifically, the broad peak at 3331 cm^−1^ is characteristic of the stretching vibration of the hydroxyl group in polysaccharides for inter- and intra-molecular hydrogen bond vibrations in cellulose. The band at ~ 2900 cm^−1^ is attributed to [ν(C–H)] stretching vibration of all hydrocarbon constituents in polysaccharides. Typical bands of cellulose are observed in the region of 1630–850 cm^−1^. The peak located at ~ 1640 cm^−1^ corresponds to the vibration of water molecules absorbed in cellulose. The bands at 1428–1429, 1367–1375, 1334, 1111, 1163, 1027 cm^−1^, and 896–897 cm^−1^ belong to stretching, bending, and scissoring vibrations of –CH_2_ and –CH, –OH, C–O–C, and C–O bonds in cellulose. The band at 1420–1430 cm^−1^ refers to the crystalline region of the cellulose, while the band at 897 cm^−1^ is attributed to the amorphous fraction (Hospodarova et al. 2018).

In the region, 3660–3200 cm^−1^ differences of hydrogen bonds in cellulose crystalline polymorphs can be reflected by absorptions of [ν(O–H)] stretching in the IR spectra. Thus, IR spectroscopy can be used for the discrimination of different polymorphs. According to the literature, cellulose Iβ was featured by a band at 3420 cm^−1^. Cellulose was characterized by bands at 3480 cm^−1^, 3440 cm^−1^, and a peak as shoulder at 3150 cm^−1^ (Hong et al. 2021). The wavenumbers 1429, 1163, 1111, and 897 cm^−1^ are indicative of cellulose crystallinity and its forms (Nelson & O’Connor 1964). The 1429 cm^−1^ band is significant in cellulose I, appearing weak with a shift to 1420 cm^−1^ in cellulose II. The band at 1163 cm^−1^ is identical in cellulose I. The shift of cellulose II is slightly to 1156 cm^−1^, compared to amorphous cellulose. The band at 1111 cm^−1^ is strong in cellulose I, exhibits reduced intensity and a shift to 1102 cm^−1^ in cellulose III and is only observed as a minor feature in the spectra of cellulose II and amorphous cellulose. At 897 cm^−1^, the band is weak and broad in cellulose I, strong and sharp in cellulose II but shifted to 893 cm^−1^, and less strong than in cellulose II (Nelson & O’Connor 1964).

Samples demonstrated the characteristic bands of cellulose. In the range of 3660–3200 cm^−1^, there are no considerable differences between the samples. The major difference between peach celluloses and the reference sample is found in the one strong band at 2900 cm^−1^ for Södra purple—attributed to cellulose I—instead of the band of the two sharp peaks at 2917 and 2848 cm^−1^ in the other spectra, which possibly suggests the co-presence of cellulose II, as a result of the treatment of the raw material with NaOH. Finally, the presence of the peak at 894.8 cm^−1^ in the case of A3B1C1 corroborates the existence of cellulose II.

Thus, all the FT-IR spectra are consistent. Similar to the XRD analysis, the results confirmed the presence of cellulose, the high purity of the samples, and the formation of cellulose II due to the treatment with NaOH.

### SEM analysis

**Fig. 12:**
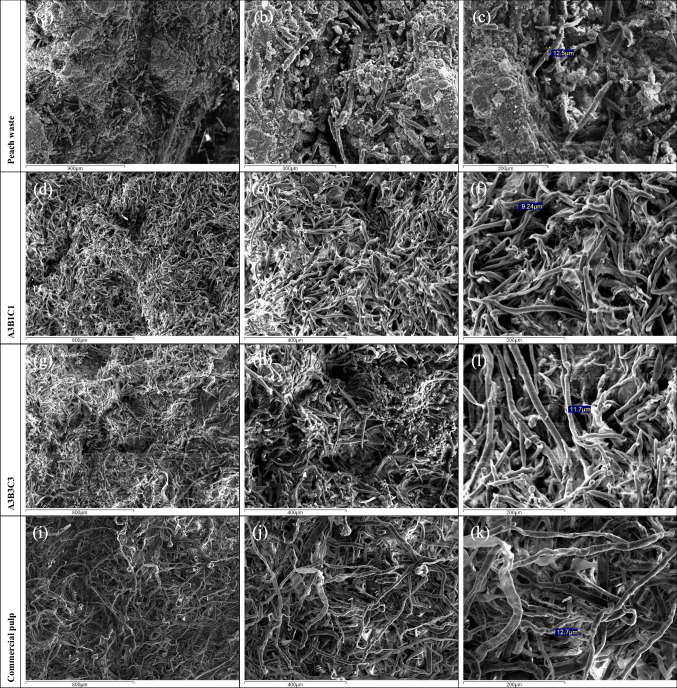
SEM imagesat different levels of magnification: × 65 (**a**), × 180 (**b**), × 250 (**c**), × 80 (**d**, **g**, **j**), × 150 (**e**, **h**, **j**), and × 300 (**f**, **l**, **k**)

SEM microscopy was used to observe the fibrils that showed the best chemical composition, A3B1C1 and A3B3C3 (high percentage of alpha-cellulose, relatively high DP). In addition, for comparison reasons, representative SEM images were taken of dried peach waste (raw material) and Södra purple, commercial dissolving pulp. The images are shown in Fig. 12. By examining Fig. 12a, b, and c of the raw material, we can see that the cellulose fibrils are attached to other not-well-defined substances. In images Fig. 12d to f, the morphology of the treated samples is fibrous, which is typical for cellulosic materials (Adeleye et al. 2022). Moreover, unwanted substances appear to be removed, which is in accordance with the already presented results of the other analysis techniques. The images of samples A3B1C1 and A3B3C3 demonstrate primarily the longitudinal section of the extracted fibrils found in the pulp, where fibrils of a length greater than 200 μm are clearly distinguished at the magnitude of × 300. The typical diameter of the fibrils, as measured in the SEM images, can be considered comparable among all four samples, with the commercial pulp showing rather larger diameter fibrils. The cross-section of the fibrils in sample A3B3C3 is also visible in the upper right part of Fig. 12h.

### TGA analysis

**Fig. 13:**
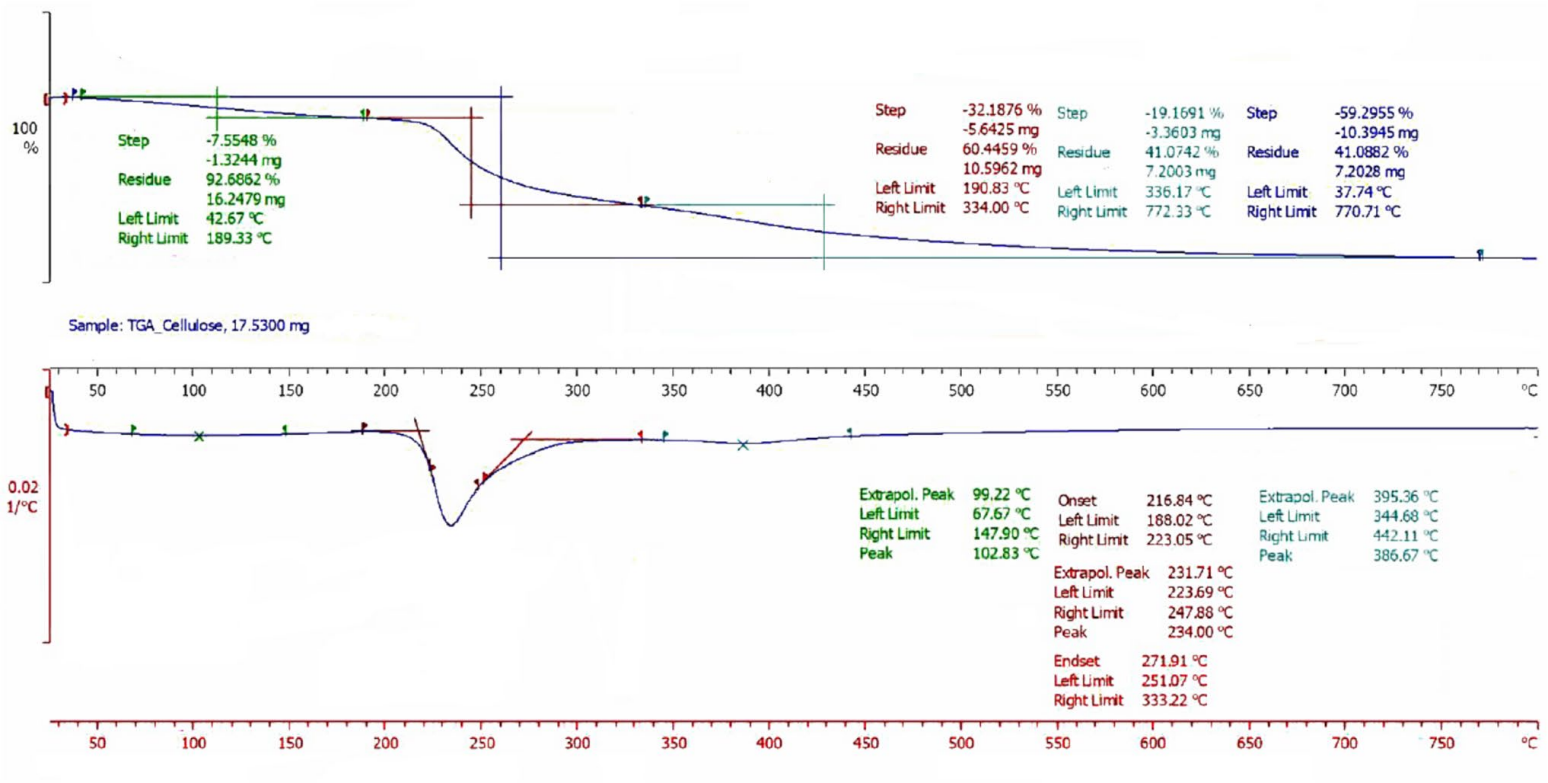
TGA graphical results—sample A3B3C3

Figure 13 demonstrates the curves of the thermostatic analysis of sample A3B3C3.

The first mass loss of the sample took place below 115 °C and can be explained by moisture evaporation. The moisture can be absorbed by cellulose during the sample handling and storage. The percentage of mass lost at this temperature corresponds to 7.55% of total mass. The main mass loss of the fibrils starts around 190 °C, ends at 334 °C, and has as a result the loss of 32.19% of the starting mass. It is related to the thermal decomposition of cellulose in these conditions and is in agreement with studies previously performed on thermal decomposition (Cheng et al. 2012; Leal et al. 2015). A third consecutive mass loss in the TGA analysis takes place between 336 and 772 °C and results in a loss of mass equal to 59.30% of the total mass. The two later steps results are expected, due to the thermal decomposition of cellulose. Cellulose decomposition involves many parallel and consecutive reactions that are affected strongly by the testing conditions, such as the atmosphere (oxidizing or inert), the heating rate, and the flow rate. It is also affected by the properties of the cellulosic material, like the crystallinity rate and the impurities (Felix et al. 2022). The third step is also associated with CO and CO_2_ formation, originated by the carbonyl groups of the cellulose molecule and the pyranose C–O–C bond, as a result of intense heating. At the end of the thermostatic, analysis test a solid carbon residue of 1.09% remained.

## Conclusions

The experimental results presented in this study establish proof of concept that high-purity cellulose pulp can be produced from peach food waste coming from the compote and juice industries. Samples A3B3C3 and A3B1C1 demonstrated satisfactory α-cellulose and DP values for use as dissolving pulp in the textile sector. The experimental series, which followed a custom fractional factorial design, revealed the strong dependence of the results on the NaOH concentration in the first stage of the process: a strong, positive, first-order correlation was found within the regression models of DP and α-cellulose, as well as a smaller, negative, second-order correlation. Thus, the middle level of NaOH delivered the best results. As expected, the whiteness of the samples correlated with the level of H_2_O_2_ used. The extraction yield ranged between 9.7 and 12.3%, but one needs to notice that there was no sorting, such as pit or other impurity removal, nor other pretreatment before the start of the process.

XRD and FTIR analysis confirmed the consistency of the patterns and bands with the dominant presence of cellulose. In addition to cellulose I, strong evidence was found for the presence of cellulose II, attributed to the use of NaOH as a cleaning and extraction agent. XRD allowed the calculation of the crystallinity degree, which ranged between 56.6 and 57.2%, which is considered very good in terms of the balance between high crystallinity, linked to mechanical strength, and low crystallinity, linked to better reactivity and dissolution capacity. SEM microscopy confirmed the morphological consistency of the produced samples with commercial dissolving-grade pulp and the accomplishment of purification by comparing the SEM images of the samples with the raw material. All analytical tests demonstrate that the isolated cellulose pulp possesses suitable characteristics and purity for future application in textiles.

This work’s significance stems from its alignment with circular economy principles, as it produces a biobased and biodegradable alternative to traditional cellulose and synthetic sources for the textile sector. Integrating this process into industrial operations will reduce waste generation and promote resource use in the agro-industrial sector. Utilizing peach waste for dissolving pulp production provides notable environmental and economic advantages over traditional wood-based methods. Unlike wood, which requires cultivation, harvesting, and often competes with other industries, peach waste is a by-product of existing processes. This eliminates the environmental burden associated with dedicated cultivation, such as water consumption, fertilizer application, and land use.

Studies, such as the life cycle assessment (LCA) conducted for wheat straw (Parayil 2023), suggest that agricultural residues can achieve superior environmental performance when used as feedstocks for dissolving pulp, provided that these residues are actual waste streams cultivated for other purposes. This is directly applicable to peach waste, which would otherwise be discarded, further reinforcing its sustainability. While no detailed LCA has yet been conducted for peach waste-based dissolving pulp, qualitative comparisons with wheat straw suggest promising prospects. Peach waste processing avoids many of the upstream emissions tied to wood cultivation and transportation. By repurposing waste streams, the production process benefits from reduced resource extraction and waste management costs. Preliminary estimates also indicate lower environmental impacts in categories like acidification and photochemical oxidant formation, assuming efficient solvent recovery and energy use.

Furthermore, this study presents a high probability for scalability, as it uses low-cost, technical-grade reagents and processes that are compatible with existing and straightforward industrial infrastructure. The mild conditions of the experimentation favor the energy profile of the process, while the implemented experimental design can easily be adjusted to provide proof of concept for the use of different agro-industrial streams. Future work should focus on pilot-scale trials to validate the process efficiency and cost-effectiveness at larger scales, as well as implementing a LCA and a life cycle costing (LCC) to assess its environmental footprint and the cost-effectiveness of the proposed process.

## Data Availability

Data will be made available on reasonable request.

## References

[CR1] Adeleye OA, Bamiro OA, Albalawi DA, Alotaibi AS, Iqbal H, Sanyaolu S, Femi-Oyewo MN, Sodeinde KO, Yahaya ZS, Thiripuranathar G, Menaa F (2022) Characterizations of alpha-cellulose and microcrystalline cellulose isolated from cocoa pod husk as a potential pharmaceutical excipient. Materials 15(17):5992. 10.3390/ma15175992 36079372 10.3390/ma15175992 PMC9457090

[CR2] Ajao O, Marinova M, Savadogo O, Paris J (2018) Hemicellulose based integrated forest biorefineries: implementation strategies. Ind Crops Prod 126:250–260. 10.1016/j.indcrop.2018.10.025

[CR3] Balkissoon S, Andrew J, Sithole B (2023) Dissolving wood pulp production: a review. Biomass Conversion and Biorefinery 13(18):16607–16642. 10.1007/s13399-022-02442-z

[CR4] Biswas MC, Dwyer R, Jimenez J, Su H-C, & Ford E (2021) Strengthening regenerated cellulose fibers sourced from recycled cotton T-shirt using glucaric acid for antiplasticization. Polysaccharides, 2(1) Article 1. 10.3390/polysaccharides2010010

[CR5] Blair MJ, Mabee WE (2021) Techno-economic and market analysis of two emerging forest biorefining technologies. Biofuels, Bioprod Biorefin 15(5):1301–1317. 10.1002/bbb.2218

[CR6] Brooks RE, Moore SB (2000) Alkaline hydrogen peroxide bleaching of cellulose. Cellulose 7(3):263–286. 10.1023/A:1009273701191

[CR7] Cheng K, Winter WT, Stipanovic AJ (2012) A modulated-TGA approach to the kinetics of lignocellulosic biomass pyrolysis/combustion. Polym Degrad Stab 97(9):1606–1615. 10.1016/j.polymdegradstab.2012.06.027

[CR8] Chiriac A, Pastor F, Popa V, Aflori M, Ciolacu D (2014) Changes of supramolecular cellulose structure and accessibility induced by the processive endoglucanase Cel9B from Paenibacillus barcinonensis. Cellulose 21:203–219. 10.1007/s10570-013-0118-x

[CR9] DemmaCarà P, Pagliaro M, Elmekawy A, Brown D, Verschuren P, Raveendran S, Rothenberg G (2013) Hemicellulose hydrolysis catalysed by solid acids. Catal Sci Technol 3:2057–2061. 10.1039/c3cy20838a

[CR10] Dritsopoulos A, Zacharopoulos N, Peyret A-E, Karampella E, Tsoureas N, Cheilari A, Machalia C, Emmanouilidou E, Andreopoulou AK, Kallitsis JK, Philippopoulos AI (2024) Ruthenium-p-cymene complexes incorporating substituted pyridine–quinoline ligands with –Br (Br-Qpy) and –phenoxy (OH-Ph-Qpy) groups for cytotoxicity and catalytic transfer hydrogenation studies: synthesis and characterization. Chemistry 6(4):773–793. 10.3390/chemistry6040046

[CR11] FAO. (2023). *FAOSTAT* . https://www.fao.org/faostat/en/#data/QCL

[CR12] Felix CB, Chen W-H, Ubando AT, Park Y-K, Lin K-YA, Pugazhendhi A, Nguyen T-B, Dong C-D (2022) A comprehensive review of thermogravimetric analysis in lignocellulosic and algal biomass gasification. Chem Eng J 445:136730. 10.1016/j.cej.2022.136730

[CR13] French AD (2014) Idealized powder diffraction patterns for cellulose polymorphs. Cellulose 21(2):885–896. 10.1007/s10570-013-0030-4

[CR14] Gehmayr V, Sixta H (2012) Pulp properties and their influence on enzymatic degradability. Biomacromol 13:645–651. 10.1021/bm201784u 10.1021/bm201784u 22300287

[CR15] Gong J, Li J, Xu J, Xiang Z, Mo L (2017) Research on cellulose nanocrystals produced from cellulose sources with various polymorphs. RSC Adv 7:33486–33493. 10.1039/C7RA06222B

[CR16] Grigelmo-Miguel N, Gorinstein S, Martín-Belloso, O. (1999) Characterisation of peach dietary fibre concentrate as a food ingredient. Food Chem 65(2):175–181. 10.1016/S0308-8146(98)00190-3

[CR17] Harsono H, Putra AS, Maryana R, Rizaluddin AT, H’ng YY, Nakagawa-izumi A, Ohi H (2016) Preparation of dissolving pulp from oil palm empty fruit bunch by prehydrolysis soda-anthraquinone cooking method. J Wood Sci 62(1):65–73. 10.1007/s10086-015-1526-3

[CR18] Hong T, Yin J-Y, Nie S-P, Xie M-Y (2021) Applications of infrared spectroscopy in polysaccharide structural analysis: progress, challenge and perspective. Food Chemistry: X 12:100168. 10.1016/j.fochx.2021.100168 34877528 10.1016/j.fochx.2021.100168 PMC8633561

[CR19] Hospodarova V, Singovszka E, Stevulova N (2018) Characterization of cellulosic fibers by FTIR spectroscopy for their further implementation to building materials. Am J Anal Chem 09(06):303–310. 10.4236/ajac.2018.96023

[CR20] Huang C, Sun R, Chang H, Yong Q, Jameel H, Phillips R (2019) Production of dissolving grade pulp from tobacco stalk through SO2-ethanol-water fractionation, alkaline extraction, and bleaching processes. BioResources 14(3):5544–5558. 10.15376/biores.14.3.5544-5558

[CR21] Hussain S, Jõudu I & Bhat R (2020) Dietary fiber from underutilized plant resources—a positive approach for valorization of fruit and vegetable wastes. Sustainability, 12(13), Article 13. 10.3390/su12135401

[CR22] Jahan MS, Rahman MM, Ni Y (2021) Alternative initiatives for non-wood chemical pulping and integration with the biorefinery concept: a review. Biofuels, Bioprod Biorefin 15(1):100–118. 10.1002/bbb.2143

[CR23] Jiang X, Bai Y, Chen X, Liu W (2020) A review on raw materials, commercial production and properties of lyocell fiber. Journal of Bioresources and Bioproducts 5(1):16–25. 10.1016/j.jobab.2020.03.002

[CR24] Kim DB, Lee WS, Jo SM, Lee YM & Kim BC (2001) Phase transition of cellulose solutions in N-methyl morpholine N-oxide hydrates. Polymer Journal 33(1):18–26. https://www.nature.com/articles/pj20014

[CR25] Kingdom FAA (2011) Lightness, brightness and transparency: a quarter century of new ideas, captivating demonstrations and unrelenting controversy. Vision Res 51(7):652–673. 10.1016/j.visres.2010.09.012 20858514 10.1016/j.visres.2010.09.012

[CR26] Kosan B, Thümmler K, Meister F, Römhild K (2024) Suitable dissolving pulps and their impacts on solution spinning of cellulose man-made fibers. Cellulose 31(3):1941–1955. 10.1007/s10570-023-05721-8

[CR27] Leal GF, Ramos LA, Barrett DH, Curvelo AAS, Rodella CB (2015) A thermogravimetric analysis (TGA) method to determine the catalytic conversion of cellulose from carbon-supported hydrogenolysis process. Thermochim Acta 616:9–13. 10.1016/j.tca.2015.07.017

[CR28] Lee C, Mittal A, Barnette A, Kafle K, Park YB, Shin H, Johnson D, Park S & Kim S (2013) Cellulose polymorphism study with sum-frequency-generation (SFG) vibration spectroscopy: identification of exocyclic CH2OH conformation and chain orientation. Cellulose, 20. 10.1007/s10570-013-9917-3

[CR29] Li J, Liu Y, Duan C, Zhang H, Ni Y (2015) Mechanical pretreatment improving hemicelluloses removal from cellulosic fibers during cold caustic extraction. Biores Technol 192:501–506. 10.1016/j.biortech.2015.06.011 10.1016/j.biortech.2015.06.011 26081626

[CR30] Li Q, Wang A, Ding W & Zhang Y (2017) Influencing factors for alkaline degradation of cellulose. BioResources 12(1):P1263–1272. 10.15376/biores.12.1.1263-1272

[CR31] Liu Z, de Souza TSP, Holland B, Dunshea F, Barrow C & Suleria HAR (2023) Valorization of food waste to produce value-added products based on its bioactive compounds. Processes 11(3), Article 3. 10.3390/pr11030840

[CR32] Llano T, Arce C, Ruiz G, Chenna N & Coz A (2018). Modelling and optimization of the last two stages of an environmentally-compatible TCF bleaching sequence. BioResources, 13(3):6642–6662. 10.15376/biores.13.3.6642-6662

[CR33] Lu F & Ralph J (2010) Lignin. In *Cereal straw as a resource for sustainable biomaterials and biofuels* (pp. 169–207). Elsevier. 10.1016/B978-0-444-53234-3.00006-7

[CR34] Ma Y, Nasri-Nasrabadi B, You X, Wang X, Rainey TJ, Byrne N (2021) Regenerated cellulose fibers wetspun from different waste cellulose types. Journal of Natural Fibers 18(12):2338–2350. 10.1080/15440478.2020.1726244

[CR35] Maphosa Y, Jideani VA (2016) Dietary fiber extraction for human nutrition—a review. Food Rev Intl 32(1):98–115. 10.1080/87559129.2015.1057840

[CR36] Matin M, Rahaman MM, Nayeem J, Sarkar M, Jahan MS (2015) Dissolving pulp from jute stick. Carbohyd Polym 115:44–48. 10.1016/j.carbpol.2014.08.090 10.1016/j.carbpol.2014.08.090 25439866

[CR37] Mendes ISF, Prates A, Evtuguin DV (2021) Production of rayon fibres from cellulosic pulps: state of the art and current developments. Carbohyd Polym 273:118466. 10.1016/j.carbpol.2021.118466 10.1016/j.carbpol.2021.118466 34560932

[CR38] Nelson ML, & O’Connor RT (1964) Relation of certain infrared bands to cellulose crystallinity and crystal latticed type. Part I. Spectra of lattice types I, II, III and of amorphous cellulose. J Appl Polym Sci 8(3):1311–1324. 10.1002/app.1964.070080322

[CR39] Nikmatin S, Irmansyah I, Hermawan B, Kardiansyah T, Seta FT, Afiah IN & Umam R (2022) Oil palm empty fruit bunches as raw material of dissolving pulp for viscose rayon fiber in making textile products. Polymers 14(15) Article 15. 10.3390/polym14153208 10.3390/polym14153208 PMC9370854 35956722

[CR40] Opperskalski S, Franz A, Patanè A, Siew S & Tan E (2022) *Preferred Fiber& Materials Market Report* (9; p. 75). Textile Exchange. https://textileexchange.org/app/uploads/2023/11/Materials-Market-Report-2023.pdf

[CR41] Pagán J, Ibarz A (1999) Extraction and rheological properties of pectin from fresh peach pomace. J Food Eng 39(2):193–201. 10.1016/S0260-8774(98)00163-0

[CR42] Parayil, M. M. (2023). *LCA of producing dissolving pulp from agricultural residue*.

[CR43] PÉrez S, & Samain D (2010) Structure and engineering of celluloses. In D. Horton (Ed.), *Advances in Carbohydrate Chemistry and Biochemistry* (Vol. 64, pp. 25–116). Academic Press. 10.1016/S0065-2318(10)64003-6 10.1016/S0065-2318(10)64003-6 20837198

[CR44] Plakantonaki S, Kiskira K, Zacharopoulos N, Belessi V, Sfyroera E, Priniotakis G & Athanasekou C (2024) Investigating the routes to produce cellulose fibers from agro-waste: an upcycling process. ChemEngineering 8(6), Article 6. 10.3390/chemengineering8060112

[CR45] Plakantonaki S, Roussis I, Bilalis D, Priniotakis G (2023) Dietary fiber from plant-based food wastes: a comprehensive approach to cereal, fruit, and vegetable waste valorization. Processes 11(5):1580. 10.3390/pr11051580

[CR46] Plakantonaki S, Stergiou M, Panagiotatos G, Kiskira K, Priniotakis G (2022) Regenerated cellulosic fibers from agricultural waste. AIP Conf Proc 2430(1):080006. 10.1063/5.0077088

[CR47] Quintana E, Valls C, Roncero MB (2024) Dissolving-grade pulp: a sustainable source for fiber production. Wood Sci Technol 58(1):23–85. 10.1007/s00226-023-01519-w

[CR48] R. Batalha LA, Colodette JL, Gomide JL, Barbosa LCA, Maltha CRA, & B. Gomes FJ (2011). Dissolving pulp production from bamboo. BioResources 7(1):640–651. 10.15376/biores.7.1.640-651

[CR49] Rojas-Valencia MN, Galeana-Olvera E, Y. Fernández-Rojas D, Mendoza-Buenrostro C, Nájera-Aguilar HA & Vaca-Mier M (2018) Isolation of cellulose nanofibrils from coconut waste for the production of sewing thread. Advanced Material Science, 3(1). 10.15761/AMS.1000135

[CR50] Rousu P, Rousu P, Anttila J (2002) Sustainable pulp production from agricultural waste. Resour Conserv Recycl 35(1):85–103. 10.1016/S0921-3449(01)00124-0

[CR51] Santanocito AM & Vismara E (2015) *Production of textile from citrus fruit* (World Intellectual Property Organization Patent WO2015018711A1). https://patents.google.com/patent/WO2015018711A1/en

[CR52] Sarkar M, Nayeem J, Popy RS, Quadery AH, Sarwar Jahan M (2018) Dissolving pulp from jute wastes. Bioresource Technology Reports 4:96–100. 10.1016/j.biteb.2018.09.008

[CR53] SAS Institute Inc. (2024). *JMP®Pro (18.0.2)* (Version 18.0.2) [Computer software]. SAS Institute Inc.

[CR54] Sayakulu NF, Soloi S (2022) The effect of sodium hydroxide (NaOH) concentration on oil palm empty fruit bunch (OPEFB) cellulose yield. J Phys: Conf Ser 2314(1):012017. 10.1088/1742-6596/2314/1/012017

[CR55] Sayyed AJ, Deshmukh NA, Pinjari DV (2019) A critical review of manufacturing processes used in regenerated cellulosic fibres: viscose, cellulose acetate, cuprammonium, LiCl/DMAc, ionic liquids, and NMMO based lyocell. Cellulose 26(5):2913–2940. 10.1007/s10570-019-02318-y

[CR56] Sixta H (2006) Pulp properties and applications. In *Handbook of Pulp* (pp. 1009–1067). John Wiley & Sons, Ltd. 10.1002/9783527619887.ch11

[CR57] Suess HU (2010). *Pulp bleaching today*. de Gruyter.

[CR58] Textile Exchange (2023). *Materials Market Report 2023*. Textile Exchange. https://textileexchange.org/app/uploads/2023/11/Materials-Market-Report-2023.pdf

[CR59] Toushik SH, Lee K-T, Lee J-S, Kim K-S (2017) Functional applications of lignocellulolytic enzymes in the fruit and vegetable processing industries: applications of lignocellulolytic enzymes. J Food Sci 82(3):585–593. 10.1111/1750-3841.13636 28152204 10.1111/1750-3841.13636

[CR60] Vera RE, Vivas KA, Urdaneta F, Franco J, Sun R, Forfora N, Frazier R, Gongora S, Saloni D, Fenn L, Zhu JY, Chang H, Jameel H, Gonzalez R (2023) Transforming non-wood feedstocks into dissolving pulp via organosolv pulping: an alternative strategy to boost the share of natural fibers in the textile industry. J Clean Prod 429:139394. 10.1016/j.jclepro.2023.139394

[CR61] Walawska A, Olak-Kucharczyk M, Kaczmarek A, Kudzin MH (2024) Environmentally friendly bleaching process of the cellulose fibres materials using ozone and hydrogen peroxide in the gas phase. Materials 17(6):1355. 10.3390/ma17061355 38541509 10.3390/ma17061355 PMC10972073

[CR62] Zanchetta E, Damergi E, Patel B, Borgmeyer T, Pick H, Pulgarin A, Ludwig C (2021) Algal cellulose, production and potential use in plastics: challenges and opportunities. Algal Res 56:102288

[CR63] Zeronian SH, Inglesby MK (1995) Bleaching of cellulose by hydrogen peroxide. Cellulose 2(4):265–272. 10.1007/BF00811817

[CR64] Zhang H, Zhang H, Tong M, Shao H, Hu X (2008) Comparison of the structures and properties of lyocell fibers from high hemicellulose pulp and high α-cellulose pulp. J Appl Polym Sci 107(1):636–641. 10.1002/app.27129

